# Next steps after 15 stimulating years of human gut microbiome research

**DOI:** 10.1111/1751-7915.13970

**Published:** 2021-11-24

**Authors:** Thomas Clavel, Hans‐Peter Horz, Nicola Segata, Maria Vehreschild

**Affiliations:** ^1^ Functional Microbiome Research Group Institute of Medical Microbiology RWTH University Hospital Aachen Germany; ^2^ Phage Biology Research Group Institute of Medical Microbiology RWTH University Hospital Aachen Germany; ^3^ Department CIBIO University of Trento Trento Italy; ^4^ Department of Internal Medicine, Infectious Diseases University Hospital Frankfurt Goethe University Frankfurt Frankfurt am Main Germany

## Abstract

Gut microbiome research has bloomed over the past 15 years. We have learnt a lot about the complex microbial communities that colonize our intestine. Promising avenues of research and microbiome‐based applications are being implemented, with the goal of sustaining host health and applying personalized disease management strategies. Despite this exciting outlook, many fundamental questions about enteric microbial ecosystems remain to be answered. Organizational measures will also need to be taken to optimize the outcome of discoveries happening at an extremely rapid pace. This article highlights our own view of the field and perspectives for the next 15 years.

## Graphical abstract

Gut microbiome research has bloomed over the past 15 years. However, many fundamental questions about enteric microbial ecosystems remain to be answered. This article highlights our own view of the field and perspectives for the next 15 years.

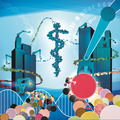

## Renewed interest in gut microbes thanks to novel technologies

**Fig. 1:**
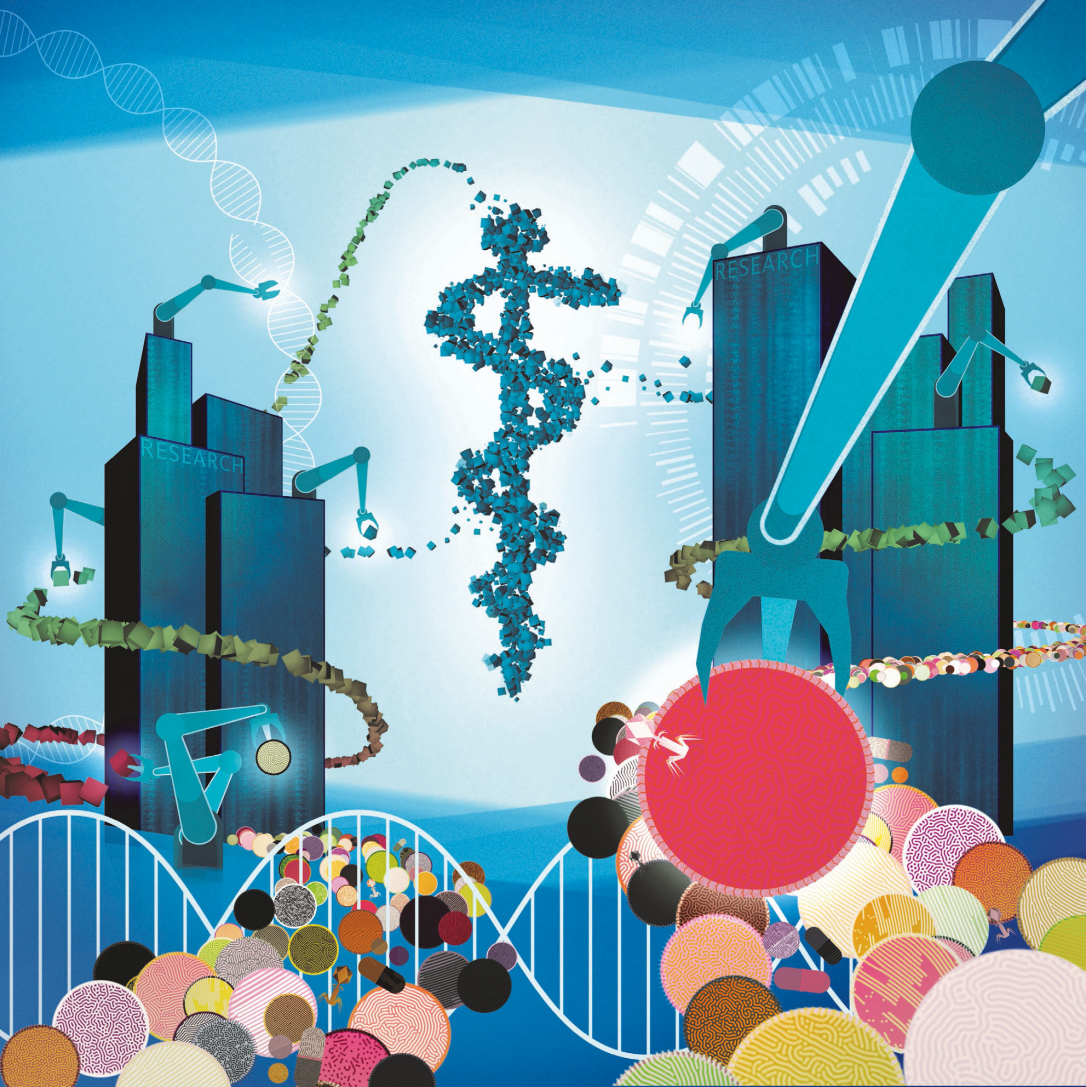
Gut microbiome research landscape.The diversity and functional potential still hidden within gut microbial communities is tremendous. Describe all microbes, their genes, proteins and metabolites by means of molecular techniques and cultivation; learn how to manipulate them as single organisms or communities; implement translational programmes in the clinics; these are exciting endeavours for the years to come. This illustration was created by Matthias Stoll.

The study of commensal microbial populations in the intestine of humans and other animals started to draw attention already in the 1960s, including the role of gut microbes in host health using gnotobiotic animal models (Dubos and Schaedler, 1960; Savage and Dubos, 1968). After discovery of the 16S rRNA molecule in the 1980s (Woese *et al*., 1980) and the advent of high‐throughput sequencing methods in the 2000s (Weinstock, 2012), gut microbiomes gained a lot of attention again during the last 2 decades. After all, these microscopic organisms influence our health and the development of infectious and chronic diseases, which is of interest to anyone (Wilkinson *et al*., 2021). Research projects on gut microbiomes have reached unprecedented scales, for example, archives of microbial diversity around the globe and sequencing one million of human metagenomes. Microbiome research is heading towards a bright future! In 15 years from now, molecular analysis of microbiomes will be implemented in clinical routines to help diagnose diseases and develop personalized pharmaceutical and nutritional therapies. All intestinal microbes (prokaryotes, fungi, viruses) and their niches in the gut will be known, and therapies based on customized synthetic communities will be possible. The way to this ideal scenario is, however, steep and with serpentines (Box 1). In other words, several fundamental steps must be taken prior to reaching this goal, some of which are detailed in the next sections (Fig. 1).

Box 1 **Challenges for the years to come in human gut microbiome research**

- Too many ecosystem members are still unknown. In 15 years from now, we will **need to know many more of them**! Knowing them all until then is too ambitious; but the current pace of description must accelerate. This concerns not only bacteria that have attracted most of the attention, but also archaea, fungi and viruses; studying the latter is most challenging.
- The focus of research needs to be further shifted from individual microbes and their role in influencing health and disease towards the **ecology within gut microbiomes**. A better understanding of fundamental rules driving interactions within gut microbial communities and the dynamics through which they are acquired, transmitted and adapted to single individuals is needed to make further progress.
- The analysis of faecal samples has dominated the field. The intestine has a very specific **biogeography** and it will be important to put more efforts in precisely understanding variations in community structure and functions longitudinally (along the tract) and transversally (from lumen to mucosa). This should be accompanied by the use of quantitative and imaging methods as opposed to the widespread concept of relative abundance determination via sequencing approaches.
- There are so many reports on disturbances of the human gut microbiomes in disease conditions. The causal role of gut microbes in disease onset and progression has been demonstrated several times. In contrast, there are far less examples of molecular mechanisms identified to be important for microbe‐host interactions. Identifying more of the specific **microbial molecules and mechanisms** regulating host pathophysiology will be an important endeavour for the next few years.
- As with all blooming fields of research, the strikingly rapid pace of reported findings in microbiome research has generated very heterogeneous results quality. To optimize and boost further progress, **community and infrastructural measures** are needed, e.g. new educational programmes for young scientists, efficient data processing and management strategies, easy access to materials (e.g. isolates) and published work. An endeavour that must include many stakeholders and should not increase the administrative burden on scientists further.

## Deeper, faster, cheaper: how much further can we go with molecular microbial ecology?

Cultivation‐free approaches have refashioned microbiome research and became first available to many researchers worldwide when high‐throughput 16S rRNA gene amplicon sequencing started to be affordable in 2007–2010 (Hamady and Knight, 2009). Nowadays, shotgun metagenomics is standard for accurate profiling of gut microbiomes (Quince *et al*., 2017), especially when analysing stool (the processing of mucosal samples is more challenging). Efficient taxonomic and functional profilers as well as assembly based approaches for metagenomics have been developed (Segata *et al*., 2012; Li *et al*., 2015; Nurk *et al*., 2017; Kang *et al*., 2019; Milanese *et al*., 2019; Beghini *et al*., 2021), which have permitted to achieve remarkable results already: the identification of disease‐ and condition‐associated biomarkers in very large datasets (Zeevi *et al*., 2015; Lloyd‐Price *et al*., 2019; Thomas *et al*., 2019; Asnicar *et al*., 2021); the characterization of single microbial strains showing that the gut microbiome of each individual is unique (Schloissnig *et al*., 2013; Franzosa *et al*., 2015; Truong *et al*., 2017; Van Rossum *et al*., 2020); the assessment of microbial transmission from mothers to infants during the first phases of life (Koenig *et al*., 2011; Ferretti *et al*., 2018; Korpela *et al*., 2018; Laursen *et al*., 2021; Walter and Hornef, 2021); the observation of thousands of yet‐uncultured and previously neglected microbial species (Almeida *et al*., 2019; Nayfach *et al*., 2019; Pasolli *et al*., 2019). However, as standardized sample preparation protocols are now available and latest cutting‐edge technologies enable sequencing at a depth of 5–10 GB for ca. USD 100 (for projects involving at least a few hundred samples), the bottleneck is now the computation of raw sequencing data and downstream interpretation (Quince *et al*., 2017).

As the costs of high‐throughput sequencing will continue to decrease, enabling the sequencing of even larger metagenomic datasets at a higher depth, computational demand will become the main limiting factor. Main challenges include the storage, processing and management of data and corresponding sample metadata as well as their effective integration across multiple studies, not to mention environmental issues associated with power‐greedy supercomputers. These challenges have no easy solution, especially given the reducing pace of computing hardware improvement and the lack of personnel trained in advanced bioinformatics. This will ultimately result in the need to trade‐off accuracy for scale in certain scenarios. Indeed, analyses of publicly available metagenomes can already reach the scale of more than 50 thousand metagenomes and collection of several million metagenome‐assembled genomes are becoming available, with no clear solution yet about how they can be efficiently stored, retrieved and analysed.

New technical breakthroughs are to be expected within the next 15 years, but their paths are difficult to predict. Long‐read and portable sequencing approaches are already being implemented (Tedersoo *et al*., 2021). If the field turns towards intensive use of such technologies, computational tools will need to be completely rethought. Moreover, when other omics (metatranscriptomics, metaproteomics and metabolomics) reach the popularity and standardization of metagenomics, they will enable a much deeper understanding of gut microbial functions, but new analytical strategies for multi‐omics data integration will be needed. It is also very likely that single‐cell technologies become more effective in surveying thousands of strains within any given microbiome, opening completely new venues in the molecular understanding of microbiomes (Hatzenpichler *et al*., 2020). All in all, the promise of microbiome‐based personalized medicine will come true only if future technological developments are coupled with substantial advances in the computational and quantitative fields (e.g. statistics).

## The renaissance of cultivation

Human gut microbiome research already offers so many promises for the near future; what would it be if the ecosystem were fully characterized! Many gut bacteria are still unknown, not to mention interactions between them. For the ones that have been cultured or we know exist, so many genes are still of unknown function or fully uncharacterized. This weakens the power of multi‐omics approaches substantially due to low annotation rates. Estimates about the cultured fraction of gut microbiomes vary depending on studies (e.g. the host species considered, and the method used for estimation). In short, we can cultivate more than a minority (30–60% of sequence‐based diversity) (Thomas and Segata, 2019; Hitch *et al*., 2021a), but a lot of work is still ahead of us. Similar to gnotobiology, pioneering anaerobic cultivation studies date back from the 1960s (Attebery and Finegold, 1969; Moore and Holdeman, 1974). However, methods of identification have greatly improved, and so many isolates have been lost over the decades due to local storage only (see the section below about ‘Infrastructural needs’). The field has now realized the need to use again anaerobic cultivation to obtain isolates; projects on the gut microbiome from different host species have been published and more are ongoing (Lagkouvardos *et al*., 2016; Seshadri *et al*., 2018; Forster *et al*., 2019; Zou *et al*., 2019; Wylensek *et al*., 2020; Groussin *et al*., 2021; Liu *et al*., 2021).

An important task for the coming years will be to develop cultivation approaches with a much higher throughput. The cultured fraction estimates mentioned above (up to 60%) refer to the global coverage of sequence‐based diversity by any bacteria isolated so far. Despite earlier work on the establishment of individual strain collections (Goodman *et al*., 2011; Faith *et al*., 2014), the depth of cultivation for any one individual sample remains low, even in recent state‐of‐the‐art studies (Groussin *et al*., 2021). In 15 years from now, we will be able to generate personalized collections of hundreds of phenotypically and genotypically fully characterized gut bacterial strains within a few weeks. New technologies and innovative workflows (e.g. based on microfluidics) will have to be developed to reach this goal. One challenge associated with this will be the ability to rapidly describe and name tens of thousands of isolates representing new taxa. There is no doubt that the current system to taxonomically define new bacteria and validate their names will have to evolve. Thereby, maintaining high‐quality standards will be of utmost importance. Cultivation and (meta)genomics will need to go hand‐in‐hand to tackle this endeavour. Initiatives such as the SeqCode (Murray *et al*., 2020) and bioinformatic resources and tools such as GTDB (Parks *et al*., 2020), GAN (Pallen *et al*., 2021), MiGA (Rodriguez *et al*., 2018), Protologger (Hitch *et al*., 2021b) and more to come, will help.

Importantly, it will not be sufficient to collect single organisms, but also crucial to intensify research on microbial interactions within communities. The ecological forces driving structural and functional dynamics within gut microbiomes have been neglected. The functions of a strain can vary greatly depending on the community it belongs to. In this context, the use of synthetic communities, that is, stable consortia of well‐defined strains, will be very valuable both as experimental models and as next‐generation probiotics (Brugiroux *et al*., 2016; Clavel *et al*., 2017; Elzinga *et al*., 2019; Albright *et al*., 2021).

Finally, while bacteria have attracted most of the attention because they are dominant members of gut microbial communities, other microbes will need to be studied more intensively to obtain a less biased view of microbe‐host interactions. For instance, archaea (e.g. methanogens) are also important ecosystem members, the diversity of which is still incomplete (Borrel *et al*., 2020; Youngblut *et al*., 2021). The role of fungi has also been highlighted (Bacher *et al*., 2019; Lemoinne *et al*., 2020; van Tilburg Bernardes *et al*., 2020). The most difficult task will be to comprehensively assess the diversity of host‐associated viromes (Virgin, 2014; Moreno‐Gallego *et al*., 2019). Among viruses, those preying on bacteria, that is, bacteriophages (or briefly phages), are of particular interest due to (i) their role as regulators of microbial communities (Shkoporov and Hill, 2019; Fujimoto *et al*., 2020; Lourenco *et al*., 2020; Spriewald *et al*., 2020), and (ii) their potential clinical use, particularly against multi‐drug resistant (MDR) bacteria (further detailed below in the section ‘Clinical microbiome‐based applications’). As in the case of bacteria, the majority of phages in the human gut have not yet been isolated. For instance in contrast to the wealth of knowledge about double‐stranded (ds) DNA‐phages, which numerically dominate phage collections and genomic databases, very few (+) single‐stranded (ss) RNA‐phages have been isolated and there is a complete absence of (−) ssRNA‐phages. This contrasts with the high diversity of RNA phages observed by metagenomics (Krishnamurthy *et al*., 2016; Callanan *et al*., 2020). A future challenge will be to decipher the specific environmental conditions that favour RNA‐ over dsDNA‐phages for the same host. Oxygen could be one major driver, as many phages may have adapted to the anaerobic lifestyle of their host in the intestine (Hernandez and Vives, 2020). Anaerobic culture conditions have so far not been extensively used for the isolation of gut‐derived phages (Hodges *et al*., 2021).

## Precision microbiome analyses

Disease‐associated disturbances in gut microbiomes have very often been expressed at the level of genera and higher taxonomic levels. This contrasts with the fact that multiple strains of a given species can drastically differ functionally (Karcher *et al*., 2020; Sorbara *et al*., 2020), albeit this is not a universal rule. It is thus obvious that common methods to characterize gut microbiomes at the molecular level must aim towards a higher resolution. Shotgun metagenomic approaches already allow precise strain‐level analyses (Karcher *et al*., 2020; Van Rossum *et al*., 2020; Hildebrand, 2021). Soon, full‐length, high‐throughput 16S rRNA gene sequencing combined with sequence variant analysis will do as well (Karst *et al*., 2021), although this works only for certain taxonomic groups. Nonetheless, it will take a while until workflows are fined‐tuned and the expertise spreads to end‐users. High‐quality, habitat‐specific atlas of molecular microbial diversity will be very helpful to help interpreting data at the strain level (Almeida *et al*., 2021). Moreover, landmark‐studies assessing the precision of bioinformatic tools used to establish strain‐level metagenomic catalogues will have to be performed using mock communities of realistic complexity (Meziti *et al*., 2021). Also, the extent to which all the sequence‐based strain diversity reported translates into functional differences of relevance for the ecosystem will have to be clarified.

Besides the need to identify all possible ecosystem members, the next years of research will also have to deal with what they can do. Well‐known gut microbial products such as propionate have shown astonishing effects in clinical settings (Duscha *et al*., 2020). The potential for applications will be manifold higher once the many secrets of yet unknown molecules from the gut microbiome are revealed. Projects studying microbiome‐drug interactions have been performed (Maier *et al*., 2018, 2021; Klunemann *et al*., 2021; Zimmermann *et al*., 2021). Work in this direction will have to be intensified, for instance via testing a higher number of strains and diversity of species and strains (including consortia) and by extending the spectrum and types of tested substances, for example, dietary compounds, host‐derived factors, specialized metabolites and small molecules (Sugimoto *et al*., 2019), including combinations thereof (Maier *et al*., 2021). Importantly, databases making detailed information of strains and all their specifications available and re‐usable must be further developed and supported (Reimer *et al*., 2019). Moreover, technologies allowing to study microbial functions and regulation thereof *in situ* (i.e. in their native environments) will be important (Hatzenpichler *et al*., 2020). Personalized microbial therapies will also be very relevant as a companion to classical medication to enhance responses to treatment (Daillere *et al*., 2016; Pryor *et al*., 2019). In summary, a lot will come from the study of reciprocal drug‐microbiome interactions in the near future.

Parallel to deciphering metabolic functions hidden within gut microbiomes, we must describe and characterize microbial molecules involved in microbe‐host interactions. The point about functional unknowns was addressed above. The work ahead of us is tremendous to tackle the unexplored world of metabolites and other potentially bioactive molecules produced by microbes (Chaudhari *et al*., 2021). A few examples exist, such as the role of *Akkermansia muciniphila* in regulating metabolic responses, with interventional applications turning into reality 15 years after discovery of this gut bacterium (Derrien *et al*., 2004; Depommier *et al*., 2019). Many more studies in this direction (identification of specific microbial molecules, underlying mechanisms and effects on the host up to clinical translation) are needed.

Finally, easily implementable protocols that allow tailored genetic manipulation of a wide variety of anaerobic gut commensals are also direly needed and will generate further breakthroughs in microbiome research (Lim *et al*., 2017; Whitaker *et al*., 2017; Guo *et al*., 2019; Ramachandran and Bikard, 2019). Being able to knockout‐specific genes in their native host is an obstacle that prevents proof‐of‐concept studies on the role of microbial functions in host health. For strain‐specific, targeted gene manipulations within bacterial communities, bio‐engineered phages equipped with gene‐editing modules (e.g. CRISPR‐Cas) will likely become a very useful toolbox (Yosef *et al*., 2017; Tagliaferri *et al*., 2020). Advances in synthetic biology will also certainly help investigating yet‐unknown gut microbial functions observed by metagenomics (McCarty and Ledesma‐Amaro, 2019).

## It is about time to deliver: Towards clinical microbiome‐based applications

The physiology of nearly all human organs is influenced by gut microbes and their products (e.g. metabolites), fuelling the hopes of patients and physicians for novel treatment approaches with a significant clinical impact. The field needs to multiply efforts to reach practices that are up to the promises of microbiome‐based diagnostics and interventional strategies using or targeting the microbes themselves.

Disturbances in gut microbial structure and functions have been associated with multiple diseases in the context of numerous studies, which have often been underpowered and not comparable, as exemplified by the association between microbiome changes and obesity (Falony *et al*., 2016; Sze and Schloss, 2016). In contrast, disease‐specific signatures are rare and should be studied further. Breakthroughs will come from both whole‐ecosystem shifts analysed by predictive computation models and quantitative measurements of specific ecosystem members. It is sound to think that microbiome analyses will soon be implemented in clinical settings for diagnostic purposes, for instance in the context of colorectal cancer (Thomas *et al*., 2019; Wirbel *et al*., 2019). Moreover, as gut microbes have been shown to influence drug metabolism and efficacy, microbiome‐based companion diagnostics is a flourishing area of research and developments (Haiser *et al*., 2013; Zimmermann *et al*., 2019; Roberti *et al*., 2020). Besides such promising outlook of microbiome applications to predict the onset and course of diseases, treatment options using or targeting the microbes themselves are hype but still in their infancy, as described below.

While the first randomized controlled trial successfully using faecal microbiota transfer (FMT) for the treatment of recurrent *Clostridioides difficile* infections (rCDI) was a great success (van Nood *et al*., 2013), no microbiota‐based treatment has been registered since its publication in 2013. This does not mean that there are no promising products in sight; however, multiple aspects were initially underestimated. The slow translation into the clinics is mostly due to regulatory hurdles. There is a controversial discussion on whether microbiota‐based treatments should be classified as substances of human origin, as drugs, or into another yet‐undefined category. In countries where they are currently classified as drugs, production must happen under Good Manufacturing Practice (GMP) conditions, which requires a considerable infrastructural investment on the side of the researchers, long before the actual research can be initiated. Furthermore, to achieve drug status, FMT products must be standardized to ensure reproducibility of quality and safety standards. Even though it is possible to define such criteria formally, it is obvious to anyone that the uniqueness of each donor’s microbiota conflicts with the demands for standardization. Strict standardization is only possible in case the final product is based on defined microbial communities, also referred to as synthetic communities (SYN), as opposed to individual faecal donations. However, this will require biotechnological developments for efficient and stable production of SYN‐products followed by testing their efficacy in direct comparison to classical FMT (Kurt *et al*., 2021). One aspect that complicates such comparisons is that both treatment response (magnitude and duration) and the engraftment of microbial strains in the intestine of recipients are individual‐specific. The same FMT or SYN product will not perform equally well in many different patients. In 15 years from now, we will be able to use bioinformatic approaches to fine‐tune SYN compositions based on recipient gut metagenomes. Combined with a universal genomic atlas of cultured isolates that will be publicly available, personalized microbial therapies adapted to individual microbial signatures will be possible. Nonetheless, this will have to be integrated into the regulatory framework for microbiota‐based treatments. Moreover, both technological and infrastructural measures supporting the isolation and characterization of gut microbes will have to happen. Furthermore, ecological interactions and ensuing consortium stability within individual SYNs will have to be considered. Such personalized approaches might facilitate the use of FMT in indications other than rCDI, such as irritable and inflammatory bowel diseases as well as hepatic encephalopathy, for which response rates are encouraging but not as impressive as in rCDI. However, this will require a much more precise understanding of disease‐specific microbiota signatures.

Besides intervention strategies based on the direct use of microbes and communities thereof, targeted modification of the gut microbiota by external factors is on the rise again. The original concept of prebiotics was coined nearly 30 years ago (Gibson and Roberfroid, 1995). The field is now moving from a one‐fits‐all approach to the selected use of a broad variety of complex carbohydrates for precision microbiome modulation (Bindels *et al*., 2015; Deehan *et al*., 2020). Concepts towards metagenome‐educated strategies for personalized nutritional interventions have been proposed (Zeevi *et al*., 2015; Kolodziejczyk *et al*., 2019; Asnicar *et al*., 2021). Applications are already being implemented due to the general high interest in maintaining or losing body weight as easily as possible and to the widespread burden of metabolic diseases and corresponding market opportunities. We will see in 15 years from now whether the field of microbiome‐based personalized nutrition can hold all the promises. It will require large‐scale, multi‐centre clinical studies to demonstrate efficacy (Htet *et al*., 2020). Moreover, the focus of human microbiome research on Westernized populations and corresponding diseases (e.g. obesity, type‐2 diabetes) has delivered biased findings on the gut microbial ecosystem. Successful research programmes based on reciprocal diet‐microbiome interactions to combat the very important worldwide issue of childhood undernutrition are being implemented (Schwarzer *et al*., 2016; Gehrig *et al*., 2019; Mostafa *et al*., 2020; Chen *et al*., 2021).

Considering the current antibiotic crisis, phage treatments have re‐gained much interest in the past 15 years, and they can also be used experimentally to modulate complex microbial communities. However, while clinicians and surgeons long for rapid official approval to help fighting deadly infections (Moelling *et al*., 2018), regulatory and legal bodies are slow, explaining the ever‐growing number of compassionate phage therapies (McCallin *et al*., 2019). Besides natural phages, those developed by genome engineering and forward genetics (Dedrick *et al*., 2019) will facilitate efficient and safe use in clinical settings. Phage engineering will leverage research and applications towards tailored modulation of the gut microbiota, which represents a major reservoir of MDR (Hsu *et al*., 2020; Tavella *et al*., 2021). We are already capable of constructing hybrid phage particles with a standard genomic backbone coupled with exchangeable tail fibres to be able to transduce any bacterial host (Yosef *et al*., 2017; Yehl *et al*., 2019). Further development of this technology in coming years will allow functional re‐programming of selected bacteria in the gut. Although phages are highly (bacterial) host‐specific, cascading events on other microbial community members affecting metabolomes and eventually (mammalian) host functions can occur (Hsu *et al*., 2019). A paradigm shift is at the front door: phages can directly interact with mammalian cells (Bichet *et al*., 2021; Bodner *et al*., 2021). Hence, future research and applications will need to integrate this tripartite system by investigating long‐term co‐existence and evolution of phages and bacteria and by exploring potential ‘off‐target’ effects of phages (Wahida *et al*., 2021).

## This is not all about science: infrastructural needs in the field

In microbial ecosystems, diversity is usually a good attribute. Similarly, as any scientific method is somewhat biased in one way or the other, it is good to nurture a certain diversity in methods and ways to implement them in different labs. However, this is without doubts that gut microbiome research has been parasitized by artefact findings due to the misuse of methods and misinterpretation of data. As implied above, diversity is meaningful and strict harmonization must not be the goal to avoid that findings are all biased the same way. Nevertheless, initiatives that help generating high‐quality protocols via comprehensive comparisons of both wet‐lab and *in silico* procedures in microbiome research will continue being needed, from sampling to data analysis (Costea *et al*., 2017; Meyer *et al*., 2021). This should be accompanied by education efforts to spread the knowledge from expert labs to a broad spectrum of end‐users.

Part of the endeavour to promote high‐quality microbiome science will be the management of an amazingly growing amount of data, especially sequencing data. This is a community effort that must be implemented at multiple levels: funding agencies (this is a costly enterprise), scientists (who should nurture long‐term over short‐term added value and community over own benefits), universities (due to the need for appropriate infrastructure, both hardware and manpower) and scientific journals (for policies that encourage proper handling of data). The real challenge is that these different layers are intricately connected: it will harm the field if journals implement very strict policies that further increase the burden on researchers and prevent them from publishing their findings if the other layers have not been addressed. Archiving systems for sequencing data already existing, but the quality of metadata is usually low, and the data are not re‐usable, or in a very limited manner. Initiatives such as the National Research Data Infrastructure financed by the German Research Foundation (DFG) will help (https://nfdi4microbiota.de).

Importance of the availability and re‐use of materials is not restricted to molecular work. Future breakthroughs in microbiome research will come from the return to cultivation work and the use of isolates. However, infrastructural measures to help facilitating the archiving and accessibility of strains are badly needed. Programmes have been launched to collect and conserve existing diversity before it is lost (Bello *et al*., 2018; Rabesandratana, 2018). Such projects also help extend our horizon to populations outside the Western world to obtain a more comprehensive landscape of gut microbiome diversity. However, international collections of isolates are chronically underfunded considering the Hercules work of having to accommodate thousands of strains isolated worldwide, including the large‐scale projects mentioned above. This badly harms the field on a mid‐ to long‐term perspective and will require multi‐layer solutions (from funding agencies to scientific journals) as mentioned above for data management.

## Conclusive remark

A lot has happened in the field of gut microbiome research during the last two decades. The journey has, however, only started. Some selected hot‐topics are present in Box 2. Promises are many in terms of potential applications, but expectations are correspondingly high. The field has gained a lot of attention, and it is now time to consolidate and deliver. An exciting time to be a microbiome scientist!

Box 2 **Hot topics**

- Colonization of the intestine (and other body habitats) after birth and during the first years of life is a crucial process with life‐long consequences for the host. A lot remains to be elucidated to obtain a comprehensive picture of what happens during this very important window of opportunities. How does colonization occur? Where do the microbes originate from, what determine their establishment within the ecosystem, and how quickly do they evolve and adapt to their new environment? What are the main ecological forces driving community dynamics and attractor states of infant gut microbial communities? How can we best intervene to modulate them for long‐term beneficial effects on the host? Research on the **infant gut microbiome** will generate important breakthroughs in the next few years.
- The near future will also see the rise of microbiome studies being performed at unprecedented scales. The development of **innovative and integrative bioinformatic approaches** to handle such data will continue being very important. At the same time, high‐end molecular work is accompanied by the renaissance of **microbiome studies based on cultivation**, which brings taxonomic and functional studies of isolates again in the forefront. In 15 years from now, we will want to know as many ecosystem members as possible and have them available. The infrastructural needs that will be required to accommodate massive amounts of data and to maintain reliable and well‐curated biological resources (e.g. biobanks) will be correspondingly high, but worth the investment.
- **Microbiome editing** will be a main topic to address in coming years. At the community level, this requires understanding major ecological factors driving community assembly and microbial interactions and being able to intervene by replenishing the ecosystem with important missing microbes (and their functions). At the strain level, there is a dire need for targeted genetic engineering of commensal microbes *in vitro* and *in vivo*. Progress in this area will generate breakthroughs in the field.
- **Translational studies** are needed. Expectations for clinical and population‐wide applications of microbiome‐based discoveries are high due to the popularity of our field. There are multiple possible tracks ahead, and it is hard to predict which ones will lead to successful outcome. As mentioned above, enhancing the power of diagnostic approaches via the gut microbiome will happen soon. It will take a little longer to implement treatments using the microbes themselves (FMT, next‐generation probiotics/postbiotics) due to regulatory hurdles and our yet‐incomplete understanding of microbial diversity and interactions. Nonetheless, multiple FMT trials in the context of *C. difficile* infection, inflammatory bowel diseases and cancer drug efficacy are ongoing. Concepts for personalized nutrition and precision medicine using stool microbiome profiles are there, but their validation and proof of efficacy will require large‐scale, multi‐centre, randomized trials. In the next 15 years, a lot will come from research on the gut‐brain axis and interactions between gut microbes and the nervous system. Hopefully, work on microbiome‐based applications to help fighting malnutrition will also have a large positive impact by then.

## Conflict of interest

TC has ongoing scientific collaborations with Cytena GmbH and HiPP GmbH and is member of the scientific advisory board of Savanna Ingredients GmbH. MJGTV received research grants from 3M, Astellas Pharma, Biontech, DaVolterra, Evonik, Gilead Sciences, Glycom, Immunic, MaaT Pharma, Merck/MSD, Organobalance, Seres Therapeutics, Takeda Pharmaceutical. She also received speaker and/or consulting fees from Alb Fils Kliniken GmbH, Arderypharm, Astellas Pharma, Basilea, Bio‐Mérieux, DaVolterra, Farmak International Holding GmbH, Ferring, Gilead Sciences, Immunic AG, MaaT Pharma, Merck/MSD, Pfizer, Roche, Organobalance, SocraTec R&D GmbH. NS reports consultancy contracts with Zoe, Roche, Ysopia and Freya and is co‐founder of PreBiomics.

## Funding information

Deutsche Forschungsgemeinschaft (DFG, German Research Foundation): Project no. 403224013 – SFB1382 and Project no.460129525 – NFDI4Microbiota

## Author contributions

TC coordinated the project. All authors wrote the manuscript and agreed with its final content.
